# Longitudinal shifts in avoidance-based phenotypes in a predator odor conditioned place aversion model of posttraumatic stress disorder

**DOI:** 10.3389/fnbeh.2026.1907963

**Published:** 2026-08-25

**Authors:** Meghan B. Reiss, Daniel K. Humphrey, Laken N. Pierceall, Michelle A. Dean, Lisa M. Tran, Rose Snijders, Lucas C. Carstensen, Dipta Chandra Pal, Dae-Wook Kang, Maria E. Secci, Nicholas W. Gilpin, Nicole M. Collette, Doris Lam

**Affiliations:** 1 Biosciences and Biotechnology Division, Lawrence Livermore National Laboratory, Livermore, CA, United States; 2 Department of Civil and Environmental Engineering, The University of Toledo, Toledo, OH, United States; 3 Department of Physiology, School of Medicine, Louisiana State University Health Sciences Center, New Orleans, LA, United States; 4 Alcohol and Drug Abuse Center of Excellence, Louisiana State University Health Sciences Center, New Orleans, LA, United States; 5 Southeast Louisiana VA Healthcare System, New Orleans, LA, United States

**Keywords:** Avoiders, behavior, Non-Avoiders, posttraumatic stress disorder, predator odor, conditioned place aversion

## Abstract

**Introduction:**

Posttraumatic stress disorder (PTSD) develops in a subset of trauma-exposed individuals and is characterized by persistent intrusion, avoidance, negative mood and cognition, and arousal symptoms, with heterogeneous symptom trajectories. There is a need for longitudinal rodent models of PTSD that incorporate individual differences in post-stress outcomes to probe mechanisms of susceptibility and resilience.

**Method:**

We used a predator odor (bobcat urine) conditioned place aversion (CPA) paradigm in male Wistar rats to examine the emergence and persistence of anxiety-like avoidance behavior over 6 weeks. Automated tracking during CPA testing quantified time in the predator odor (PO)-paired chamber and chamber-specific locomotor metrics; animals were further assessed with an acoustic startle response (ASR) test (Day 2), open field (OF, Day 5), and elevated plus maze (EPM, Day 16). At 6 weeks post-stress, rats were re-exposed to the CPA apparatus, modeling contextual reminder-induced trauma recall. Longitudinal body weight and endocrine endpoints (adrenal gland weight and circulating corticosterone) were collected.

**Results:**

At 24 h post-stress, Avoider rats, approximately 40% of exposed animals, showed avoidance of the PO-paired chamber, whereas Non-Avoider rats made more entries into and moved more slowly within the PO-paired chamber. By 6 weeks, initial classifications no longer predicted avoidance behavior; CPA-based diagnosis revealed substantial phenotypic shifts, with 24% of rats remaining Avoiders, 28% remaining Non-Avoiders, and the remainder switching phenotypes (16% Avoiders to Non-Avoiders; 32% Non-Avoiders to Avoiders). Re-analysis of the ASR test showed similar startle responses across phenotypes at lower and intermediate intensities; however, rats that shifted from Non-Avoiders to Avoiders displayed greater startle responses than rats that remained Non-Avoiders at the highest sound level. In contrast, OF and EPM measures showed no phenotypic differences. Sustained Avoider rats exhibited modestly greater weight gain, whereas corticosterone and adrenal gland mass did not differ among groups.

**Discussion:**

These findings indicate that PO models identify early Avoider and Non-Avoider phenotypes with distinct behavioral profiles but also reveal dynamic shifts in avoidance-based classifications over time. This work highlights the need for longitudinal studies to better characterize evolving behavioral phenotypes, endocrine, and physiological changes in rodent PTSD models and understand neural mechanisms underlying interindividual variability in PTSD symptom trajectories.

## 1 Introduction

Posttraumatic stress disorder (PTSD) is a complex and chronic neuropsychiatric disorder resulting from a traumatic experience. Numerous psychological and physical factors have been shown to increase the likelihood of developing and maintaining PTSD, such as natural disasters, assault, sexual violence and abuse, combat and war zone stressors, and serious injury (*e.g.*, traumatic brain injury). Meta-analysis of these factors show variability in the prevalence of PTSD resulting from a single event, ranging from below 1% for natural disasters and up to 7.3% for physical and sexual assault (Kilpatrick et al., 2013), approaching 30% in conflict-affected regions (Ahmed et al., 2024; Kilpatrick et al., 2013), and <8% in the general population (Kilpatrick et al., 2013; Koenen et al., 2017). The Diagnostic and Statistical Manual of Mental Disorders, 5th edition (DSM-5) requires that individuals exposed to a qualifying traumatic event exhibit intrusion (reexperiencing) symptoms, persistent avoidance of trauma-related internal or external stimuli, negative alterations in cognition and mood, and marked alterations in arousal and reactivity. These symptoms must persist for more than 1 month after the trauma exposure and cause clinically significant distress or impairment (American Psychiatric Association, 2013).

In recent years, longitudinal clinical studies have highlighted considerable heterogeneity in the course of PTSD, with distinct symptom trajectories that include persistently elevated, persistently low, increasing, or decreasing symptom severity following trauma (Bonde et al., 2022; Brier et al., 2020). Most trauma-exposed individuals show natural recovery and do not develop chronic PTSD, yet the mechanisms underlying resilience and delayed or persistent symptomatology remain poorly understood. Consequently, distinguishing individuals who will develop chronic PTSD versus those who will recover is a major challenge in this field.

Animal models remain valuable for probing mechanisms that underlie PTSD, especially when they can accommodate individual differences in posttraumatic outcomes and offer a manner with which they may be examined as variables. In such models, only a subset of animals develops persistent maladaptive responses after stress, allowing stratification into affected/Avoider (Avoider) and unaffected/Non-Avoider (Non-Avoider) phenotypes. This approach enhances the face validity of preclinical models and better captures core PTSD-relevant phenotypes potentially analogous to those observed in humans (Albrechet-Souza and Gilpin, 2019; Cohen et al., 2004; Verbitsky et al., 2020). However, most rodent PTSD paradigms assess behavioral and neurobiological outcomes within the acute or early post-stress period (days to weeks), largely focusing on immediate or early responses to trauma and potential “windows of opportunity” for early intervention (Carmi et al., 2016; Verbitsky et al., 2020; Yamamoto et al., 2009). By contrast, there are a relatively limited number of studies that extend assessment into the ≥1 month timeframe normally required for a clinical PTSD diagnosis (Edwards et al., 2013; Mackenzie et al., 2010; Mancini et al., 2021; Wilkinson et al., 2023). This gap underscores the need for preclinical models that examine the chronicity and trajectory of PTSD-relevant symptoms.

In the present study, we used a predator odor (PO) (bobcat urine) conditioned place aversion (CPA) rat model to generate Avoider and Non-Avoider phenotypes, hereafter referred to as “Avoider” and “Non-Avoider.” This model is ethologically relevant for rodents and produces PTSD-relevant avoidance behaviors that parallel key aspects of human symptomatology, fulfilling 4 of 5 key criteria for a valid animal model of PTSD (Albrechet-Souza and Gilpin, 2019; Whitaker and Gilpin, 2015; Yehuda and Antelman, 1993). Avoider rats, which comprise approximately 35% of Wistar rats (with the remaining 65% being Non-Avoiders), exhibit robust, stable avoidance and anxiety-like behavior (Albrechet-Souza and Gilpin, 2019; Albrechet-Souza et al., 2020, 2021). Here, we assessed predator odor avoidance behavior and its persistence over 6 weeks following CPA by using acoustic startle response (day 2 post-stress), open field (day 5 post-stress), and elevated plus maze (day 16 post-stress) tests; we then retested predator odor-paired chamber avoidance at 6 weeks post-stress to evaluate the stability of Avoider and Non-Avoider phenotypes during contextual reminder induced trauma recall. This study identified subsets of animals that may exhibit acute phenotypes that do not persist, and delayed phenotypes that can be identified at late assessment time points, suggesting heterogeneity in assessments that only consider early post-stress time points.

## 2 Materials and methods

All procedures with animals adhered to the Office of Laboratory Animal Welfare (OLAW) guidelines and regulations at Lawrence Livermore National Laboratory, including ethics review and Institutional Animal Care and Use Committee (IACUC) approval (24-09-082). Animals were housed in an AAALAC-accredited, PHS-assured, laboratory animal facility.

### 2.1 Animals

Seventy-two male Wistar rats were obtained from INOTIV (Livermore, CA) and used in this study. Animals were 200–275 g (approximately 5–9 weeks old) upon arrival and weighed 257.3 ± 14.9 g (mean body weight ± SD) at the start of the experiment, when they were approximately 6–10 weeks old. Animals were pair-housed in static microisolator cages with irradiated sterilized bedding, sterile paper shred nesting material, consumable shelter-type enrichment, and resting lofts under a reversed 12-h light/12-h dark cycle (lights off at 9:30 am) and were given *ad libitum* access to irradiated sterile standard rodent chow (Teklad 2918) and water. Male animals were chosen for this study to maintain statistical power to address the primary question of longitudinal phenotype persistence and avoid an underpowered two-sex design. The effect sizes established here will inform appropriately powered, follow up studies that include both sexes.

### 2.2 Experimental design and behavioral assessments

**FIGURE 1:**
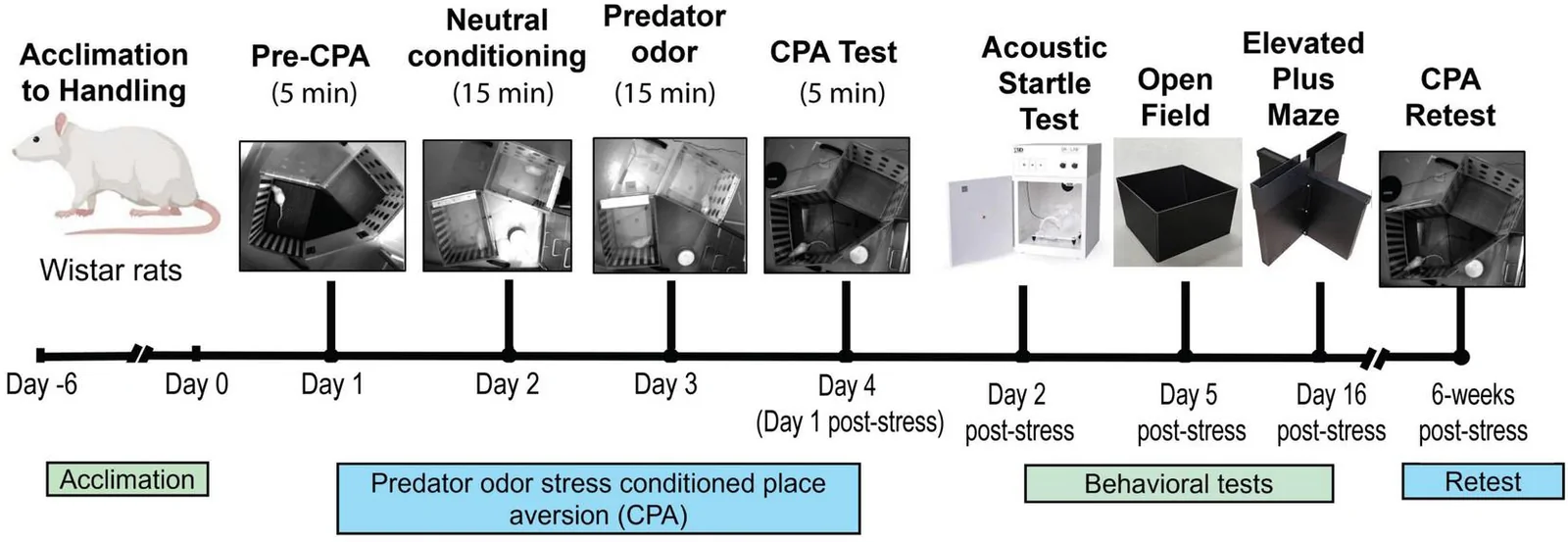
Experimental design. The schematic illustrates the experimental design used to evaluate the effects of PO on the persistence of PTSD relevant symptoms in rats. Animals were handled for a week prior to the 4-day CPA procedure. Following CPA, rats completed a battery of behavioral tests including unconditioned acoustic startle test, open field, and elevated plus maze. At 6 weeks post-stress, rats were re-tested in the CPA apparatus to re-expose them to the trauma context. Terminal blood samples and tissues were collected within 24 h following the re-test session. Created in BioRender. Lam, D. (2026) https://BioRender.com/pv9jr0o. Publication license: Institution - Academic; Agreement No. YL2A1PIZK8; Publisher: Frontiers in Behavioral Neuroscience.

This study followed a 6-week experimental regimen (Figure 1) where all experiments were conducted during the rats’ dark cycle. Rats were given 1 week to acclimate to their surroundings and were handled daily prior to the initiation of the experimental procedures. On the day of each behavioral procedure/test, rats were weighed at Day 0 (handling/acclimation to the test room), Day 1, Day 2, Day 3, and Day 4 of the conditioned place aversion (CPA) procedure, as well as Day 2, Day 5, Day 16, and 6-weeks post-stress. Day 1 commenced the CPA procedure. The analysis of weight gained over the course of the experiment was determined by the differences between 6-weeks post-stress and Day 3 of the CPA-procedure divided by 41 days. On Day 3, rats were exposed to bobcat urine, an etiologically validated stressor that can effectively induce a range of behavioral and physiological responses following a single exposure (Albrechet-Souza and Gilpin, 2019). Control rats followed the same CPA procedure but were given no odor. In this study, 24 h post-stress (Day 4 of the CPA procedure), predator odor (PO)-exposed rats were indexed as “Avoiders” or “Non-Avoiders” based on the calculated CPA score, consistent with previous studies that identified rats as “Avoiders” and “Non-Avoiders” (Albrechet-Souza and Gilpin, 2019; Albrechet-Souza et al., 2020; Edwards et al., 2013; Weera et al., 2023; Whitaker et al., 2014), respectively. Rats completed a battery of behavioral tests using time points and tests that have previously shown their sensitivity in detecting differences between the Avoider versus Non-Avoider phenotypes (Albrechet-Souza et al., 2020; Edwards et al., 2013; Roltsch et al., 2014; Weera et al., 2023; Figure 1). This included acoustic startle tests on Day 2 post-stress, open field on Day 5 post-stress, elevated plus maze on Day 16 post-stress, and re-test to the trauma-paired chamber of the CPA apparatus at 6-weeks post-stress. The combination of behavioral tests performed at specific time points was designed to evaluate interindividual stress response and its persistence over the 6-week period, to confirm face validity of this model in reproducing the persistent anxiety-related symptoms characteristic of PTSD.

#### 2.2.1 Predator odor exposure

Rats underwent a classical 4-day CPA paradigm, as described in previous studies (Edwards et al., 2013; Weera et al., 2023; Whitaker and Gilpin, 2015). The CPA apparatus consisted of two chambers, each measuring 15″ (length) × 12″ (width) × 15″ (height), separated by a triangular platform with 15″ sides. All testing was conducted under indirect dim illumination (∼10 lux per chamber) and ∼70 dB white noise. The apparatus was thoroughly cleaned and dried between animals and at the end of the procedure with CaviWipes (Metrex, Orange, CA), a quaternary ammonium and alcohol-based surface disinfectant. After testing, a mint-based deodorant, PureAyre (Clean Earth, Inc., Kent, WA) was used to wipe apparatus surfaces and fogged into the test room. On Day 1, each rat underwent a 5-min video-recorded pre-test, which allowed them to freely explore two distinct conditioning chambers. These chambers were different in tactile (circles versus square mesh flooring) and visual (stripes versus circles) cues. Chamber assignments for rats were randomized. On Day 2, each rat was placed in one chamber for 15 min without odor (neutral environment). On Day 3, each rat was placed in the opposite chamber for 15 min with a paper towel soaked with 15 mL bobcat urine (cat# 91412 PP, Maine Outdoor Solutions, Herman, ME), positioned under the floor of the stress exposure chamber (PO environment), and the lid to the chamber was closed for the exposure period. Fresh bobcat urine was applied to the paper towels and left to soak for 15 min in between each pair of rats being exposed. On Day 4, rats underwent a 5-min video-recorded test to explore both chambers. Control rats followed the same procedure but were not exposed to PO.

##### 2.2.1.1 Automated video tracking for the CPA procedure

We employed an automated video tracking system, ANY-maze (Wood Dale, IL), to increase throughput of independent tests which allows post-recording analysis of up to 8 video recordings simultaneously. Zones were created in ANY-maze for each of the two chambers, the adjoining center triangle, and zones representing the walls of the apparatus. Results for the entire apparatus included measures for total distance traveled, average speed, and total number of freezing episodes (defined as lack of movement for >1 s). Potential freezing episodes were flagged by ANY-maze, and validated manually. Freezing was defined as the complete absence of all voluntary movement, except for respiration (Fanselow, 1980), and was scored by two blinded raters who independently reviewed the video recordings at the time points flagged by ANY-maze. In addition, features analyzed for the defined predator odor-paired chamber included the distance traveled, total number of entries into the chamber, average speed within the chamber, and freezing episodes within the chamber. Entry into any of the three zones required 40% of the animal’s body to cross from another zone, with the exception of the neutral center zone, which used ANY-maze’s automated entry detection based on the center point of the animal. Visualization of test results were generated using track plots of the rat’s trajectory and heat maps indicating time spent in each location.

The CPA score was determined by calculating the difference in time spent in the odor-paired chamber during the test compared to the pre-test. Rats that displayed >10 s decrease in time spent in odor-paired context on test day (Day 4 of CPA) compared to pre-test day (Day 1 of CPA) were classified as “Avoiders.” Rats that displayed a <10 s decrease in time spent in odor-paired chamber on test day compared to pre-test day were classified as “Non-Avoiders.” This classification criterion was based on previous findings indicating that a 10-s cut-off reliably distinguishes persistent avoidance or “Avoider” behaviors predictive of subsequent brain and behavioral changes (Albrechet-Souza and Gilpin, 2019; Edwards et al., 2013; Whitaker et al., 2014).

#### 2.2.2 Acoustic startle response (ASR) test

Rats were assessed for unconditioned ASR on Day 2 post-stress, as previously described (Albrechet-Souza et al., 2020; Roltsch et al., 2014). Each rat was placed in a Plexiglass tube connected to an accelerometer within a dark, soundproof chamber (San Diego Instruments, San Diego, CA) and allowed to acclimate for 5 min prior to testing. The test session consisted of 30 trials, with startle stimuli of three different white noise intensities (90 dB, 105 dB, and 115 dB), each randomly presented 10 times, and separated by a fixed 30-s intertrial interval. Each trial involved a 750 ms burst of white noise. The plexiglass tube was cleaned with a paper towel to remove any excrement, disinfected with CaviWipes, before wiped again with a paper towel and fully air dried in between subjects. The startle response was recorded as maximum movement velocity (Vmax) during the first 100 ms of each trial, with one measurement every millisecond. The primary dependent variable, average ASR, was calculated as the mean of these 30 trials. Data were reported as the average ASR normalized to the rat’s body weight at the time of testing.

#### 2.2.3 Open field

The open field test was used to examine anxiety-like behavior at Day 5 post-stress, as previously described (Whitaker and Gilpin, 2015). The open field was fabricated in-house using a black Acrylonitrile Butadiene Styrene (ABS) plastic. The arena (black ABS, 100 cm × 100 cm) was placed in the center of a dimly lit room. Each rat was placed in the center and allowed to explore for 5 min while behavior was recorded; the experimenter was not present during testing. The apparatus was cleaned with disinfectant CaviWipes between subjects. A blinded observer analyzed videos using ANY-maze. The arena was divided into a center zone (25% of total area) and edge zone (75%) by overlaying a 10 cm × 10 cm grid; the four central squares defined the center, and the remaining squares defined the edge. Zone entries were detected automatically by ANY-maze based on body position. Measures for the entire arena included total distance traveled, average speed, total freezing episodes, and time spent freezing. Zone-specific measures included entries, distance traveled, and average speed. Percent time in the center was calculated as: s in center/(s in center + s in edge) × 100, and percent time freezing as: s freezing/300 s of recording × 100.

#### 2.2.4 Elevated plus maze

As described before (Albrechet-Souza et al., 2020), the elevated plus maze (EPM) test was used as an additional measure of anxiety-like behavior at Day 16 post-stress. The EPM (San Diego Instrument, San Diego, CA) was made from ABS and consisted of two closed arms (19.75′′ long × 4′′ wide) with a wall height of 12′′, two open arms (19.75′′ long × 4′′ wide), and an open center (4′′ × 4′′). The maze was 19.5′′ off the floor in the center of the room, and each arm had similar levels of illumination. The test was initiated by placing the rat in the open center of the EPM and behavior was recorded for 5 min. The observer was not present in the room during testing. The EPM was cleaned thoroughly with the disinfectant, CaviWipes, between subjects. A blinded observer analyzed the video recordings using ANY-maze, creating the boundaries for the open and closed arms. One entry was defined as 85% of body area entering the open/closed arm, and 70% of the animal’s body had to remain in the zone for it to be counted as within the zone. Parameters analyzed included time spent freezing, total number of freezing episodes, total distance traveled and average speed. Zone-specific measures collected (for both the open and closed arms) included time spent in each zone, distance traveled in each zone, average speed in each zone, and entries into each zone. Percentages were calculated for % of time spent in the open arm [(s in open arm/300) × 100] and % of entries into the open arm [(entries into the open arm/entries into the open arm + entries into the closed arm) × 100].

### 2.3 Euthanasia and tissue collection

Rats were euthanized at the terminal (6 weeks post-stress) timepoint using carbon dioxide inhalation overdose followed by exsanguination and terminal blood collection according to AVMA Guidelines. After post-mortem cardiac perfusion with phosphate-buffered saline containing heparin (10 IU/mL), adrenal glands were dissected and weighed.

### 2.4 Enzyme-linked immunosorbent assays (ELISAs)

Circulating corticosterone was measured from terminal (6 weeks post-stress) blood samples. Blood was collected in K2-EDTA-containing blood collection tubes and centrifuged at 3400 rpm for 15 min at 4 °C. Plasma was collected and stored at −80 °C until needed. Commercially available ELISAs for corticosterone were performed in duplicate and conducted based on the manufacturer’s instructions (Corticosterone ELISA kit, prod. No. ADI-901-097, Enzo Life Sciences, Inc., Farmingdale, NY). Plasma samples were in the detectable range for the corticosterone (32–20,000 pg/mL), when diluted 40-fold using buffers provided by the kit. ELISA plates were read with Synergy HTX Multimode Reader set to 450 nm for the corticosterone ELISA, with correction between 570 and 590 nm. All samples were normalized to the control samples of their respective cohorts and expressed as fold-change.

### 2.5 Principal component analysis (PCA)

Principal component analysis (PCA) was performed to examine multivariate patterns and inter-individual variability across behavioral and physiological measures. Two separate feature sets were prepared for PCA analysis: (i) CPA features collected at Day 1 post-exposure and 6 weeks post-stress and (ii) a combined dataset including measures from CPA, acoustic startle response, open field test, elevated plus maze, body weight, and corticosterone. Only numeric variables were included, and diagnostic group labels were not used to compute the principal components. All variables were standardized using z-score normalization (mean = 0, standard deviation = 1), and animals with missing values across features were excluded (Jolliffe and Cadima, 2016). Group-level differences in multivariate profiles were assessed using permutational multivariate analysis of variance (PERMANOVA) based on Euclidean distances with 999 permutations. Non-parametric pairwise PERMANOVA comparisons between diagnostic groups were conducted (Anderson, 2001). To further examine group differences along individual PCA dimensions, Mann–Whitney U tests were applied to PC1 and PC2 scores. All analyses were carried out in RStudio (2023.09.1) using R (v4.5.0, with PCA implemented *via* the *prcomp* function and PERMANOVA conducted using the adonis2 function from the vegan package (v2.7-2).

### 2.6 Statistical analysis

Quantified data are expressed as mean ± standard error of the mean (SEM) for the number of rats indicated unless stated differently. For behavior experiments, the statistical significance was analyzed in GraphPad version 10 (GraphPad Software, San Diego, CA). Comparisons between predator odor-exposed phenotypes (Avoiders *versus* Non-Avoiders) were analyzed using unpaired *t*-tests. One-way ANOVA was used to compare three or more experimental groups. Repeated measures two-way ANOVA was used for analyses involving repeated measurements within the same animal (*e.g*., acoustic startle responses across sound intensities), with sound intensity as the within-subject factor and experimental group as the between-subject factor. Standard two-way ANOVA was used where appropriate to evaluate the effects of two independent variables (*e.g.*, body weight and experimental groups). When ANOVA revealed a significant main effect or interaction, Tukey’s *post hoc* analysis was used to compare individual group means. Statistical significance was defined as *p* < 0.05.

## 3 Results

### 3.1 Automated behavioral metrics in the predator odor chamber

**FIGURE 2:**
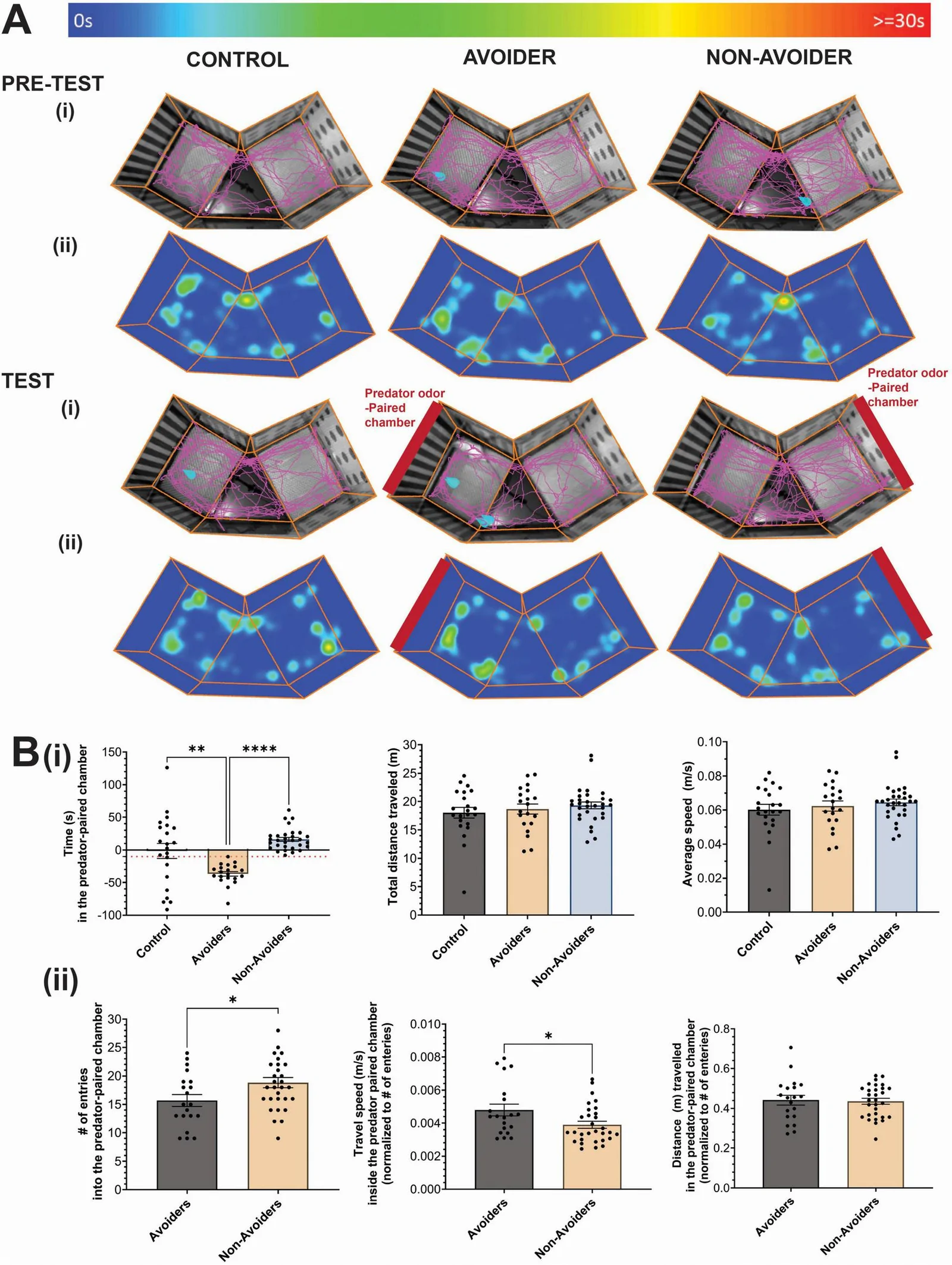
Automated tracking analysis of the CPA procedure. **(A)** Representation of control, Avoider, and Non-Avoider rat behavior in the CPA apparatus is visualized in the track plot **(i)** and respective heat map **(ii)**, for both the pre-test and test days. Movement tracks are shown in magenta. A blue tear drop indicates the location where the rat froze. For heatmaps, the brightest area (rainbow scale, red = maximum), represents the location where the animal spends the most time. The PO-paired chamber is indicated with a red bar on the outer chamber wall. **(Bi)** On Day 4 of the CPA procedure, the phenotype was determined from the CPA score, which is the difference in time spent in the PO-paired chamber in test compared to pre-test. Avoider rats were determined to avoid the PO-paired chamber (spend at least 10 s less in the PO-paired chamber), and Non-Avoider rats did not avoid the PO-paired chamber, with a cutoff value of 10 s less in the PO-paired chamber to distinguish from Avoiders (red dotted line, left bar graph). The average time (mean ± SEM s) spent in PO-paired context during the post-conditioning test (Day 4 of CPA) compared to the pre-conditioned (Day 1 of CPA) is shown in the bar graph for rats classified as Avoiders (*n* = 20) and Non-Avoiders (*n* = 30). Measures for gross locomotor activity included total distance traveled (middle graph) and average speed (right graph) during the 5-min CPA recording, are shown. **(Bii)** For metrics within the PO-paired chamber, comparisons were made between the odor-exposed rats (*e.g.*, Avoider *versus* Non-Avoider phenotype). This included the number of entries (left panel), and the travel speed (middle panel) and distance traveled (right panel), normalized to the number of entries into the PO-paired chamber. Data is shown as mean ± SEM for control (*n* = 22), Avoiders (*n* = 20), and Non-Avoiders (*n* = 30) with statistical significance detected at a level of *p < 0.05, **p < 0.01, and ****p < 0.0001.

We applied automated tracking to the CPA procedure during both the pre-test (Day 1) and post-conditioning (Day 4) tests (see also Figure 1 for experimental design and time point reference). This approach generated qualitative data, such as track plots (Figure 2Ai) and heat maps (Figure 2Aii), as well as quantitative measures, including time spent in the PO-paired chamber and additional features (Figure 2Bi) reported here for the first time. These features include gross locomotor activity within the apparatus (total distance traveled and average speed) and specific behaviors within the PO-paired chamber (number of entries, average speed, and travel distance). One day post-stress to bobcat urine, rats were classified based on their calculated CPA scores and avoidance behavior. CPA scores were calculated as the difference between the time spent in the PO-paired chamber before and after challenge with predator odor. A cutoff value of 10 s less spent in the PO-paired chamber was used to distinguish groups. Figure 2Bi presents the average CPA scores for rats in the control group (no odor exposure), as well as for those identified as Avoiders and Non-Avoiders following predator odor exposure. Individual rats in the control group displayed an inherent preference for either the stripe or circle chamber; however, the group as a whole did not show a significant preference for the designated sham PO-paired chamber (Figure 2Bi), consistent with previous findings (Weera et al., 2020). This demonstrated that, in the absence of predator odor, the chamber itself did not elicit avoidance behavior, which confirmed the control group as a valid baseline. In contrast, rats classified as Avoiders spent, on average, 36.56 ± 3.50 s less time (mean ± SEM) in the predator odor-paired chamber compared to their pre-test values, whereas Non-Avoider rats spent, on average, 16.25 ± 3.00 s more time in the PO-paired chamber compared to their pre-test values, demonstrating that the avoidance behavior in the PO-exposed groups is specifically related to the stressor and not the chamber itself. Based on the cut-off criterion applied 24 h after exposure, 40% of the PO-exposed rats (20 of 50) were classified as Avoiders of the chamber associated with the PO stressor, whereas 60% were classified as Non-Avoiders.

Quantitative analysis of the CPA procedure revealed no differences in gross locomotor activity (*e.g.*, total distance traveled and average speed) within the entire apparatus between control and PO-exposed phenotypes, indicating that overall mobility was unaffected by the stressor (Figure 2Bi, middle and right graph). However, when comparing Avoider and Non-Avoider rats, Non-Avoiders exhibited a greater number of entries into the PO-paired chamber (Figure 2Bii, left graph). When accounting for number of entries into the PO-paired chamber, Non-Avoider rats showed reduced travel speed compared to Avoider rats without a difference in the distance traveled within the PO-paired chamber (Figure 2Bii, middle graph), however there was no significant difference between groups in the distance traveled inside the PO-paired chamber (Figure 2Bii, right panel), which suggests differences in behavioral strategies or movement patterns when re-exposed to the trauma-paired environment one day post-stress. A Grubb’s Test for Outliers of human-validated freezing data showed only two animals that froze during the Day 4 of the CPA procedure. Both were statistically significant outliers, and were thus removed from the dataset (*G* = 4.477 and *G* = 4.249 for the animal(s) from the Control and Avoiders group, respectively). Interrater reliability between human validators of ANY-maze freezing data using a Pearson correlation was *r* = 0.988 for Day 4 of the CPA procedure and *r* = 0.686 for 6 weeks post-stress time point.

### 3.2 Longitudinal stability of anxiety across behavioral tests based on PTSD phenotype diagnosis 1 day after stressor

In the current study, we evaluated the persistence of anxiety-like and PTSD-relevant symptoms using a battery of behavioral tests (*i.e.*, acoustic startle test, open field, and elevated plus maze) between the initial and re-test CPA test sessions at 6-weeks post-stress. Previous work had shown that an early post-stress window (*e.g.*, 2 days post-stress) differentiated predator odor stressed male rats from unstressed control rats, but did not differentiate between Avoider and Non-Avoider rats (Albrechet-Souza et al., 2020; Roltsch et al., 2014). As expected, the startle responses varied according to sound intensity (Supplementary Figure 1A). Two-way repeated measures ANOVA (sound level × phenotype) of acoustic startle reflex responses revealed a significant main effect of sound level, *F*(1.581, 107.5) = 155.3, *p* < 0.0001. However, there were no significant main effects of the phenotype, *F*(2, 68) = 0.95, *p* = 0.39, nor a significant sound level × phenotype interaction, *F*(3.163, 107.5) = 1.02, *p* = 0.39, demonstrating that startle responses did not differ between control and PO-exposed animals, and the effect of sound level was consistent across all groups. Significant subject effects were observed, *F*(68, 136) = 3.76, *p* < 0.0001, reflecting individual variability in startle responses. These results suggested that acoustic startle reflex was robustly modulated by sound intensity, but not by phenotype.

When we examined anxiety-like behavior at Day 5 post-stress using the open field (Supplementary Figure 1B), a one-way ANOVA revealed no main effect of phenotype on percent time spent in the center, *F*(2, 69) = 0.29, *p* = 0.75. Other measures of activity and potential anxiety-like behavioral traits also revealed no statistically significant differences amongst groups (Supplementary Figure 1B). Similarly, no main effect of phenotype was observed for the percentage spent in the open arms of the elevated plus maze at 16 days post-stress, *F*(2, 68) = 1.26, *p* = 0.74 (Supplementary Figure 1C). No other measures collected from the elevated plus maze revealed a main effect of phenotype (Supplementary Figure 1C). Raw data reported as mean ± SEM for open field measures can be found as Supplementary Table 1 and for the elevated plus maze as Supplementary Table 2.

#### 3.2.1 Detection of shifts in predator odor phenotypes and reassessment of phenotypes across behavioral tests

Re-test of rats to the CPA apparatus at 6 weeks post-stress showed that rats classified as Avoiders or Non-Avoiders at day 1 post-stress were not significantly different in the time spent in the PO-paired chamber at 6 weeks (Supplementary Figure 2A), either between phenotypes or relative to controls. There were no significant differences in gross locomotor activity (*e.g.*, total distance traveled or average speed within the CPA apparatus among groups, Supplementary Figure 2B), which was consistent with findings at 1-day post-stress (Figure 2Bi). Additionally, when comparing Avoider and Non-Avoider phenotypes, no significant differences were observed in the number of entries into the PO-paired chamber, distance traveled, or travel speed within the chamber (Supplementary Figure 2C). Only one chronic study had shown that Avoider rats maintained avoidance of the context-dependent chamber at 6 weeks (Edwards et al., 2013). However, in that study no behavioral testing was conducted between the initial test and the 6-weeks re-test. This procedural difference may account for the discrepancy with the current findings, in which behavioral testing occurred between the initial test and the 6-weeks retest and may have introduced one or more confounding variables (see section “4 Discussion”). A new 6-weeks CPA score was calculated for each animal as the difference between the time spent in the PO chamber at the 6 W timepoint and the Day 1 timepoint.

**FIGURE 3:**
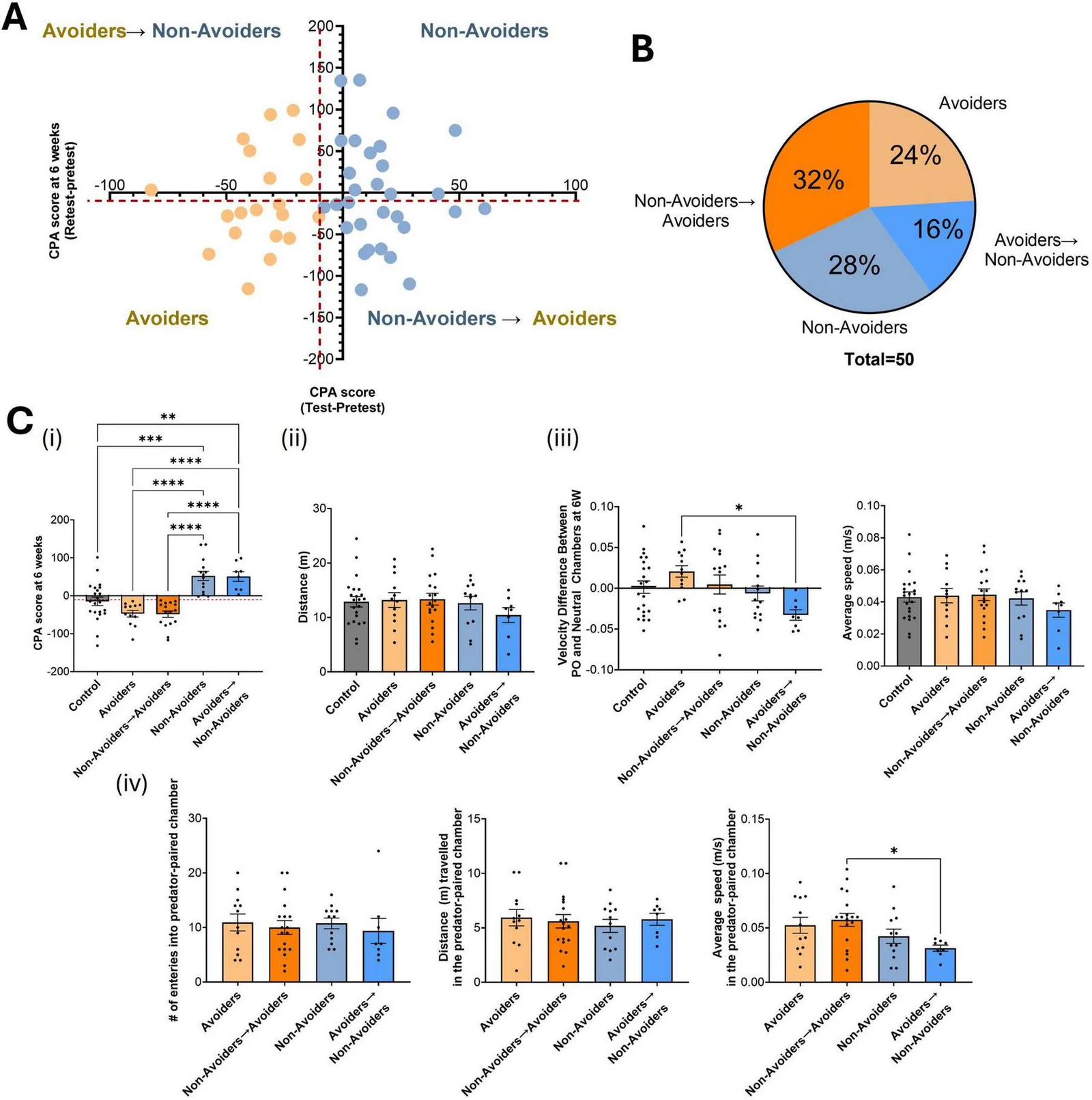
Assessment of PTSD phenotypes upon re-test to the predator odor-paired chamber at 6 weeks post-stress. **(A)** Scatter plot Avoiders and Non-Avoiders comparing Day 1 tests and 6 weeks re-tests. Red dotted line indicates the –10 s cut off score for classification of the PO phenotypes at Day 1 (*y*-axis) and 6-weeks post-stress (*x*-axis). **(B)** Pie chart displays the percentage of rats exposed to the predator odor (*n* = 50) with persistence or changes in the PTSD phenotype over the 6-weeks period. **(Ci)** The bar graphs show the mean ± SEM time spent in the PO-paired chamber (CPA score) during the re-test at 6-weeks post-stress based on re-classification of the rat’s phenotype from Day 1 and 6-weeks post-stress diagnosis. Red dotted line indicates the –10 s cut off score for classification of the PO phenotypes. **(Cii)** Total distance traveled at 6 weeks post-stress. (**Ciii**, left panel) Difference in velocity between PO-paired and neutral context is calculated as the average speed in the neutral chamber subtracted from the average speed in the PO chamber (Speed in PO-paired chamber – Speed in neutral chamber). A negative result indicates slower movement in the PO chamber relative to the neutral chamber. (**Ciii**, right panel) Average speed at 6 weeks post-stress. **(Civ)** Chamber metrics for the PO-paired context include number of entries, distance traveled, and travel speed. Data are shown as mean ± SEM for control (*n* = 22), Avoiders (*n* = 12), Non-Avoiders → Avoiders (*n* = 16), Non-Avoiders (*n* = 14), and Avoiders → Non-Avoiders (*n* = 8) groups. Statistical analysis was conducted using one-way ANOVA followed by Tukey’s *post hoc* analysis. Statistical significance is shown at a level of *p < 0.05, **p < 0.01, ***p < 0.001, ****p < 0.0001.

When we analyzed the rats’ collective CPA scores (comparing the scores obtained at 1 day and 6 weeks post-stress), using the −10 s criterion for diagnosis (red dotted line, Figure 3A), we found that a subset of PO-exposed rats shifted phenotypes, which may have contributed to the inconsistency of our findings for the time spent in the PO-paired chamber at 6 weeks post-stress compared to previous studies. Representative track plots (Supplementary Figure 3i) and heatmaps (Supplementary Figure 3ii) visualize our assessment of phenotypic shifts. The prevalence data, shown in a pie chart (Figure 3B), indicate that 8 out of 20 initially Avoider rats became Non-Avoiders at 6-weeks (16% out of 50 rats in total), 12 out of 20 rats maintained an Avoider phenotype (24%), 16 out of 30 initially Non-Avoider rats became Avoiders over the 6-weeks period (32%), and 14 out of 30 rats remained Non-Avoiders (28%). By overlaying the original diagnosis with the 6 W one, we arrived at a collective phenotype diagnosis, which holistically incorporates the data obtained at both timepoints into one label. For example, with this label, an animal that was an Avoider at D4 and a Non-Avoider at 6 W would be labeled “Avoider → Non-Avoider.” When re-analyzing the CPA re-test session at 6 weeks post-stress using the collective phenotype diagnosis at Day 1 and 6 weeks post-stress, the average CPA scores at 6 weeks are shown with additional groups for shifted phenotypes (Figure 3Ci). Quantitative analysis of gross locomotor activity again showed no difference between control and PO-exposed phenotypes over the course of 6 weeks (Figures 3Cii, iii). However, in contrast to Day 1 post-stress (Figure 2ii), the number of entries into the PO-paired chamber was no different between PO-exposed phenotypes that were sustained or had shifted (Figure 3Civ) when re-tested at 6 weeks post-stress. However, Avoiders that changed to Non-Avoiders at the 6-weeks re-test showed a significant change in velocity in the PO-paired chamber compared to rats that remained Avoiders, which may be the basis for the phenotype switch at 6-weeks post-stress. In our re-analysis, we did not normalize the travel distance and speed in the PO chamber to the number of entries into the PO chamber, as there was no difference in the number of entries into the chamber. While there was no difference in the distance traveled in the PO-paired chamber for the PO-exposed phenotypes, we did see a significant difference in the speed for the shifted rats (*e.g.*, Non-Avoider → Avoider rats *versus* Avoider → Non-Avoider rats). At the 6-weeks post-stress time point, Shapiro–Wilk tests indicated that freezing data were non-normally distributed in all phenotypes except the Avoiders → Non-Avoiders group, for both total freezing episodes and time spent freezing. For total freezing episodes, significant deviations from normality were observed in Control (*W* = 0.5397, *p* < 0.0001), Non-Avoiders (*W* = 0.3819, *p* < 0.0001), Non-Avoiders → Avoiders (*W* = 0.5233, *p* < 0.0001), and Avoiders (*W* = 0.4097, *p* < 0.0001), but not in Avoiders → Non-Avoiders (*W* = 0.8773, *p* = 0.1776). Similarly, for time spent freezing, Control (*W* = 0.5192, *p* < 0.0001), Non-Avoiders (*W* = 0.3630, *p* < 0.0001), Non-Avoiders → Avoiders (*W* = 0.4123, *p* < 0.0001), and Avoiders (*W* = 0.4328, *p* < 0.0001) showed non-normal distributions, whereas Avoiders → Non-Avoiders did not (*W* = 0.8337, *p* = 0.0649). Accordingly, nonparametric analyses were conducted using a Kruskal-Wallis test followed by Dunn’s *post hoc* multiple comparisons test. There were no significant differences between animals’ amount of freezing or time spent freezing (Supplementary Figure 4).

**FIGURE 4:**
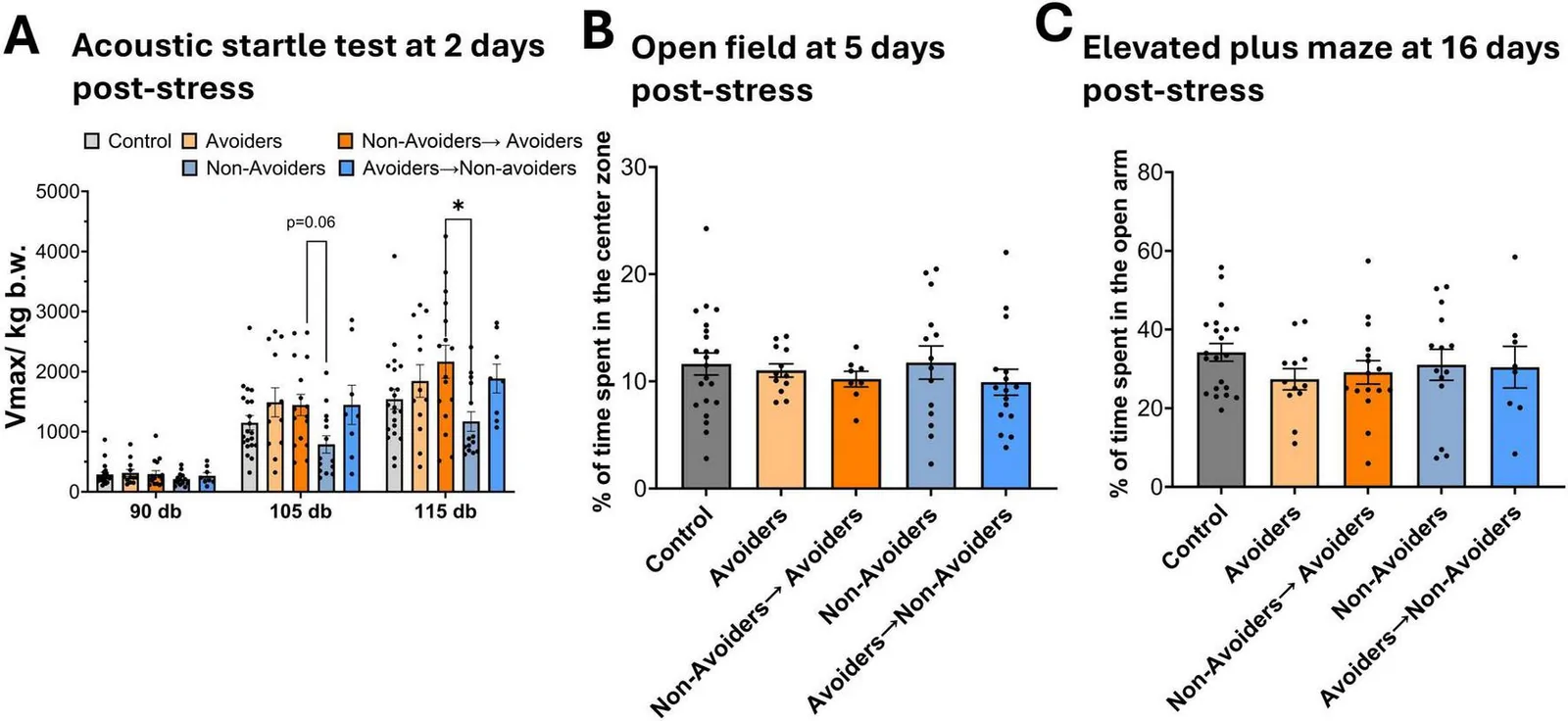
Evaluation of acoustic startle response by phenotype diagnosis at 1 day and 6 weeks post-stress. **(A)** Acoustic startle response is measured as Vmax per kilogram of body weight, for rats grouped according to PTSD phenotype at 1 day and 6 weeks post-stress. Responses are shown for each sound intensity: 90 dB, 105 dB, and 115 dB. The same groups of animals were evaluated for anxiety-like behavior in the open field **(B)**, measured as percent time spent in the center zone at 5 days post-stress, and in the elevated plus maze **(C)**, measured as percent time spent in the open arms at 16 days post-stress. Data are shown as mean ± SEM for control (*n* = 22), Avoiders (*n* = 12), Non-Avoiders → Avoiders (*n* = 16), Non-Avoiders (*n* = 14), and Avoiders → Non-Avoiders (*n* = 8) groups. Statistical comparisons were performed using one-way ANOVA with Tukey’s *post-hoc* test. Statistical significance is indicated at *p < 0.05.

To further explore whether longitudinal changes in avoidance phenotypes were associated with earlier behavioral responses, we performed an exploratory *post hoc* analysis in which animals were stratified according to their longitudinal avoidance trajectories (Avoider → Avoider, Avoider → Non-Avoider, Non-Avoider → Avoider, and Non-Avoider → Non-Avoider). Re-analysis of the acoustic startle test using the longitudinal avoidance trajectories (Figure 4A *vs*. Supplementary Figure 1A) showed that most pairwise differences between phenotypes were not statistically significant, indicating broadly similar startle responses across groups at lower and intermediate intensities. At 105 dB, the Non-Avoiders → Avoiders group showed a trend toward a greater startle response compared with the Non-Avoiders group (adjusted *p* = 0.0556), and this difference reached statistical significance at 115 dB (adjusted *p* = 0.0349). This pattern suggests that transitions from a Non-Avoider to an Avoider phenotype may be associated with heightened hyperarousal, particularly under the highest acoustic challenge. No other pairwise differences were statistically significant at any sound level. When we examined anxiety-like behavior at Day 5 post-stress using the open field (Figure 4B *vs.* Supplementary Figure 1B) and at Day 16 post-stress using the elevated plus maze (Figure 4C *vs.* Supplementary Figure 1C), a one-way ANOVA revealed no main effect of phenotype on percent time spent in the center of the open field, and similarly no main effect of phenotype on percent time spent in the open arms of the elevated plus maze. These findings suggest that, at the time points tested, these assays were not sensitive enough to detect differences between the unstressed, shifted, and sustained phenotypes.

### 3.3 Assessment of stress-related biomarkers

**FIGURE 5:**
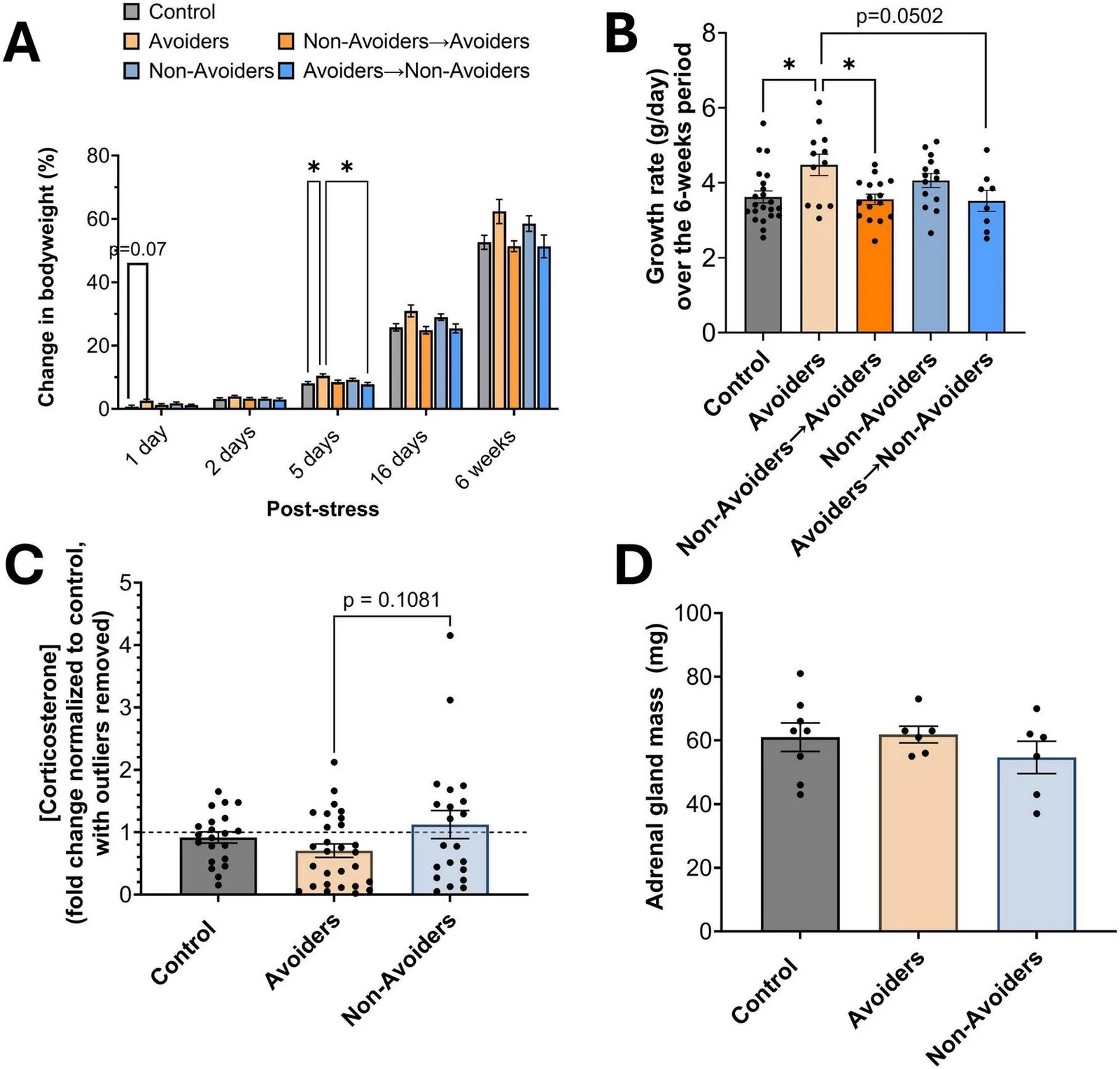
Stress-related biomarkers. Bar graphs summarize **(A)** the percentage change in bodyweight over the 6-weeks study period in sustained and shifted PO-exposed phenotypes. Data were analyzed by two-way ANOVA followed by Tukey’s *post-hoc* test; statistical significance is indicated at *p < 0.05. **(B)** Growth rate (g/day), the difference in weight at 6 weeks post-stress *versus* Day 3 of the CPA procedure is normalized to the 6-weeks period (41 days) of the study was calculated for each phenotype. **(C)** Plasma corticosterone levels are expressed as fold change relative to control animals within the same cohort; no significant differences were detected between control and PO-exposed phenotypes. Two outliers were removed from the original dataset using the Grubb’s Test for Outliers, *a* = 0.05. **(D)** At 6-weeks post-stress, adrenal glands were collected from a subset of animals (*n* = 20) and weighed. Because the shifted phenotypes were represented by fewer than three rats, adrenal mass was compared only among animals classified as control (*n* = 8), Avoiders (*n* = 6), or Non-Avoiders (*n* = 6) at 6 weeks post-stress, regardless of whether the phenotype shifted or was sustained over the course of the study. For panels **(A–C)**, data were analyzed by one-way ANOVA followed by Tukey’s *post-hoc* test with statistical significance indicated at *p < 0.05. All data are presented as mean ± SEM.

Here, we compared the percent body weight change over the 6-weeks post-stress period for sustained and shifted PO-exposed rats at time points (1 day, 2 days, 5 days, 16 days, 6 weeks) where behavioral testing was conducted (Figure 5A). There was a robust, main effect of time on percent body weight gain [time: *F*(1.161, 77.78) = 1759, *p* < 0.0001], confirming that all animals showed substantial weight gain across the 6-weeks period which is consistent with young, growing animals. In addition, both the main effect of phenotype and the time × phenotype interaction reached significance [phenotype: *F*(4, 67) = 3.922, *p* = 0.0064; time × phenotype: *F*(4.643, 77.78) = 2.619, *p* = 0.0339], indicating that growth trajectories differed modestly across phenotype groups and that these differences were time dependent. At 1 day and 2 days post-stress, no pairwise comparisons between phenotypes reached significance (all adjusted *p* > 0.05). By 5 days post-stress, only sustained Avoider rats exhibited greater percent body weight gain compared to both controls (*p* = 0.0377)and Avoider → Non-Avoider animals (*p* = 0.0272) (Figure 5A). At 16 days and 6 weeks post- stress, no comparison between phenotypes or control were significantly different in the change of body weight, although several showed trends toward statistical significance. We also examined overall growth rate across the full 6-weeks period differed by phenotype. Average daily weight change [Growth rate (g/day) over the 6-weeks study period was significantly affected by phenotype [*F*(4, 67) = 3.853, *p* = 0.0071] (Figure 5B). Specifically, the sustained Avoider rats exhibited a greater average daily weight change compared to both control and Non-Avoider → Avoider rats, whereas Non-Avoiders and shifted phenotypes (Non-Avoiders → Avoiders and Avoiders → Non-Avoiders) showed growth rates that did not differ significantly from either controls or Avoider rats. Grouped together, these findings indicate that behavioral avoidance in this model is associated with a modest increase in daily weight gain post-stress that is evident around 5 days post-stress.

Next, we assessed whether these phenotype-specific differences in body weight and growth were accompanied by alterations in Hypothalamic-Pituitary-Adrenal (HPA) axis output. In prior work, this has been observed early after stress exposure as blunted circulating corticosterone levels, with no changes in adrenal gland weight (Albrechet-Souza et al., 2020; Weera et al., 2020). In a subset of animals (*n* = 20), adrenal glands were collected and weighed. Plasma corticosterone levels were determined from blood collected at the 6-weeks terminal timepoint using ELISA (Enzo Biochem Inc.), and no significant differences were found between phenotypes (Figure 5C). Because the shifted phenotypes were represented by fewer than three rats, adrenal mass was compared only among animals classified as control, Avoiders, or Non-Avoiders at 6 weeks post-stress, regardless of whether the phenotype shifted or was sustained over the course of the study, and this analysis revealed no significant differences in adrenal mass among these three phenotypes (Figure 5D). Together, these findings suggest that, despite modest phenotype specific differences in long term weight gain, terminal corticosterone concentrations or adrenal gland mass did not differ among phenotypes at 6 weeks post-stress.

**FIGURE 6:**
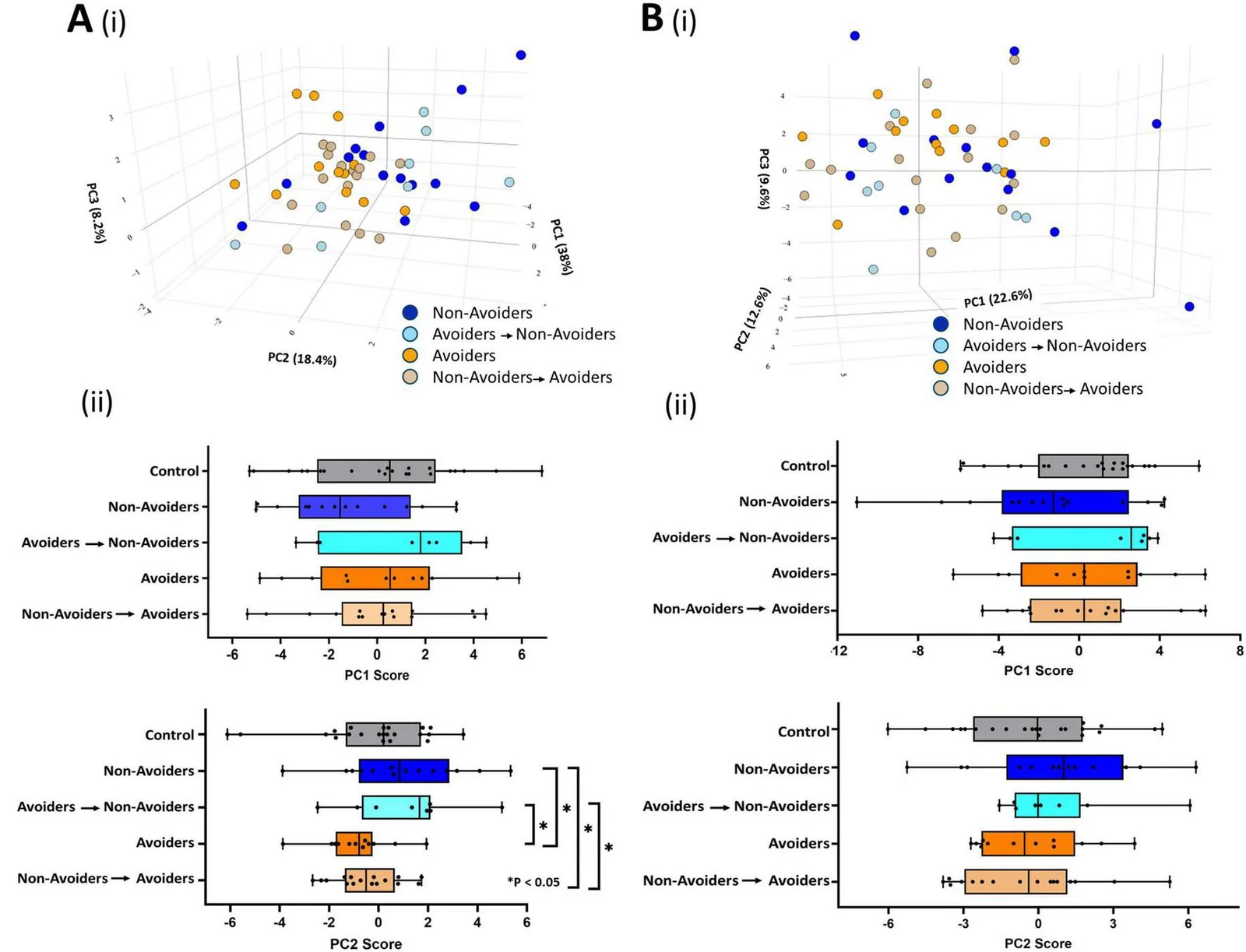
Principal component and multivariate analysis of behavioral and physiological features across diagnostic groups. **(A)** Behavioral features of CPA at Day 1 post-exposure and 6 weeks post-exposure. **(i)** Three-dimensional PCA plot (PC1–PC3) showing the distribution of individual animals based on CPA-related behavioral features, with points colored by 6-weeks diagnostic group (Non-Avoiders, Avoiders, Non-Avoiders → Avoiders, and Avoiders → Non-Avoiders). **(ii)** Horizontal box-and-whisker plots of PC1 and PC2 scores across diagnostic groups, with individual data points overlaid; significant pairwise differences identified by Mann–Whitney U tests are indicated (*p < 0.05). **(B)** Combined behavioral and physiological features. **(i)** Three-dimensional PCA plot constructed using combined behavioral (CPA, open field test, elevated plus maze, and acoustic startle) and physiological (body weight and corticosterone) variables, colored by diagnostic group. **(ii)** Corresponding box-and-whisker plots of PC1 and PC2 scores across groups, showing absence of significant pairwise differences. PCA was performed on z-score–normalized variables, and group labels were used for visualization only. The number of animals in the 6-weeks diagnostic groups were as follows: Avoiders (*n* = 12), Non-Avoiders → Avoiders (*n* = 16), Non-Avoiders (*n* = 14), and Avoiders → Non-Avoiders (*n* = 8).

Unlike the univariate behavioral analyses presented in Figure 4, principal component analysis (PCA) was used to integrate CPA-related behavioral features measured at Day 1 and 6 weeks post-exposure (Figure 6Ai), enabling assessment of the overall multivariate relationships among predator odor avoidance classifications. The first three principal components explained a substantial proportion of the total variance (PC1 = 38%, PC2 = 18.4%, PC3 = 8.2%). Pairwise PERMANOVA analyses identified significant differences between Non-Avoider and Non-Avoider → Avoider groups (R^2^ = 0.090, *p* = 0.025) and between Non-Avoiders and Avoiders groups (R^2^ = 0.112, *p* = 0.014). In addition, Non-Avoider → Avoider and Avoider → Non-Avoider groups differed significantly (R^2^ = 0.107, *p* = 0.039). No significant differences were detected in comparisons involving control animals (all *p* > 0.05). To assess whether these multivariate differences were reflected along individual PCA axes, Mann–Whitney U tests were performed on PC1 and PC2 scores across diagnostic groups (Figure 6Aii). PC1 scores did not show significant differences between groups, but PC2 scores showed significant differences between specific group pairs (Non-Avoiders *versus* Avoiders, *p* = 0.031; Non-Avoiders *versus* Non-Avoiders → Avoiders, *p* = 0.047; Avoiders *versus* Avoiders → Non-Avoiders, *p* = 0.031; Avoiders → Non-Avoiders *versus* Non-Avoiders → Avoiders, *p* = 0.052). These findings are consistent with the PERMANOVA results, which indicated that separation among CPA-related phenotypes was primarily driven by variation along the PC2 axis.

To determine whether physiological measures improved discrimination among predator odor avoidance classifications, PCA was also performed on the combined behavioral and physiological dataset including CPA, acoustic startle response, open field test, elevated plus maze, body weight, and corticosterone. Greater overlap among diagnostic groups was observed compared with CPA-only analysis above (Figure 6Bi). The first three principal components explained a moderate proportion of variance (PC1 = 22.6%, PC2 = 12.6%, PC3 = 9.6%), with no clear visual separation between groups. However, pairwise PERMANOVA identified significant differences between Non-Avoider and Non-Avoider → Avoider groups (R^2^ = 0.072, *p* = 0.023) and between Non-Avoider and Avoider groups (R^2^ = 0.078, *p* = 0.035). All other pairwise comparisons, including those involving control animals, were not significant (*p* > 0.05). Mann–Whitney U tests on PC1 and PC2 scores from the combined PCA revealed no significant differences between diagnostic groups (Figure 6Bii), suggesting that although PERMANOVA detected significant multivariate differences, these effects were distributed across multiple dimensions rather than driven by any single principal components.

## 4 Discussion

In the present study, we replicated the early post-stress response to a previously-published PO CPA model (Albrechet-Souza and Gilpin, 2019). We identified 40% of rats as Avoiders, based on avoidance of the PO-paired chamber, and 60% as Non-Avoiders, and did not avoid the PO-paired chamber. Building on prior work, we incorporated automated video tracking to quantify locomotor behavior in the PO-paired chamber, which provided additional insight into the Non-Avoider phenotype and suggested distinct strategies for navigating the PO-paired chamber that warrant further investigation. A key novel finding in our longitudinal study was the detection of predator-odor avoidance phenotypes by 6 weeks post-stress, a feature not previously reported in the PO CPA model. This finding was based on a *post hoc* analysis entailing the scoring of animals at a re-test of avoidance of the PO-paired chamber, using time spent in the PO-paired chamber as the main metric, at 6 weeks post-stress. Using sustained *versus* shifted phenotype classifications, a battery of behavioral tests (acoustic startle, open field, and elevated plus maze) were re-analyzed posthumously to assess the persistence and evolution of anxiety-related behavior. Here, we found that the acoustic startle test was sensitive to detecting changes in anxiety levels of rats that shifted from Non-Avoiders to Avoiders, exhibiting greater startle at the highest intensity compared to rats which remained Non-Avoiders. In contrast, open field and elevated plus maze behavior did not differ across phenotypes at the examined time points. Time series analysis of body weight emerged as a stress-related biomarker capable of distinguishing a sustained Avoider population, although endpoint measures of corticosterone or adrenal gland mass at 6 weeks post-stress did not show enough sensitivity as a biomarker within this model. Together, these results suggest that the PO CPA model can identify early Avoider and Non-Avoider phenotypes with distinct avoidance and Non-avoidance profiles, while also uncovering dynamic changes in phenotypes over time that complicate simple, static classifications. These findings emphasize the importance of longitudinal, multimodal behavioral assessment to precisely define evolving PTSD-relevant phenotypes in rodents and clarify neural mechanisms underlying interindividual variability in symptom trajectories.

Predator odor models have long been used in the study of innate fear, stress, and avoidance behavior in rodents. Among the early studies that helped establish the predator odor paradigms, the Blanchard group characterized defensive behavioral responses, contextual conditioning, and altered association in corticosterone and hypothalamic-pituitary-adrenal (HPA) axis function following predator odor exposure (Blanchard et al., 1998, 2001). Building on these foundational studies, the conditioned place avoidance (CPA) procedure has since become a well-established Pavlovian model for assessing stress-induced avoidance behavior and individual differences in stress susceptibility in rodents (Albrechet-Souza and Gilpin, 2019; Edwards et al., 2013). This core feature of PTSD, where an aversive stimulus is paired with a discrete environmental context, further results in persistent, individual differences in avoidance (Albrechet-Souza et al., 2020; Roltsch et al., 2014). Predator odor, typically derived from a natural predator, reliably elicits innate fear responses in both rats and mice. Bobcat urine is commonly used as the aversive stimulus in CPA paradigms. Most prior PO CPA studies have focused on time spent in the PO–paired chamber as the primary behavioral outcome. In the bobcat urine CPA framework developed by Gilpin and colleagues, animals are further classified as Avoiders or Non-Avoiders using a −10 s cutoff score (Albrechet-Souza and Gilpin, 2019; Edwards et al., 2013; Whitaker and Gilpin, 2015). Consistent with this approach, the prevalence for Avoiders and Non-Avoiders aligned with prevalence ranges reported in earlier studies (Albrechet-Souza and Gilpin, 2019). Beyond this established metric, our findings highlight the value of automated behavioral tracking for revealing additional features of behavior within the PO–paired context. Notably, Non-Avoider rats entered the PO–paired chamber more frequently and moved at slower speeds, despite traveling similar total distances compared to Avoider animals. These behavioral patterns suggest that resilience may be characterized not simply by the absence of avoidance, but by distinct behavioral strategies or patterns of movement organization, as well as other behavioral alterations, within an aversive context (Albrechet-Souza and Gilpin, 2019; Blanchard and Blanchard, 1989). Future studies using more advanced approaches (*e.g.*, machine learning and other computational tools) may further explain these behavioral strategies and determine whether they reflect enhanced risk assessment, exploratory control, or adaptive modulation of locomotion, therefore providing a deeper understanding of Avoider and Non-Avoider phenotypes in PO-based CPA models.

Contextual conditioning, or evaluation of context re-test, is a core component of behavioral phenotyping in the CPA procedure. In this approach, animals are returned to the trauma-associated environment in the absence of predator odor to assess whether the context alone elicits a conditioned response (*e.g.*, avoidance or non-avoidance of the predator odor–paired chamber). This assessment is typically conducted 24–48 h following the stressful event (*i.e.*, PO exposure) (Albrechet-Souza and Gilpin, 2019; Blanchard et al., 2001). In contrast, evaluation of conditioned behavior at longer post-stress intervals has been reported in only a couple of studies. For example, Mackenzie et al. (2010) demonstrated that a single exposure to cat odor produces long-lasting conditioned behavioral alterations, including suppressed locomotion, heightened acoustic startle, and reduced social interaction, elicited by re-test to the odor-associated cue alone for up to 28 days post-stress. Relevant to the current study, work using the bobcat urine-paired context demonstrated that a subset of animals exhibited persistent avoidance of the PO-paired context for 6 weeks post-stress, suggesting long-lasting contextual conditioning (Edwards et al., 2013). An unexpected outcome of the current longitudinal study was the emergence of phenotypic shifts by 6 weeks post-stress. Rats initially classified as either Avoiders or Non-Avoiders at Day 1 post-stress no longer differed in their average time spent in the PO-paired chamber at the 6-weeks time point (Supplementary Figure 2). However, *post hoc* reapplication of the −10 s cutoff criterion at 6 weeks post-stress revealed that distinct subgroups exhibited delayed vulnerability or recovery that were not apparent at the early post-stress assessment. Specifically, over the 6-weeks period, 24% of rats maintained an Avoider phenotype and 28% remained Non-Avoiders, whereas 16% of initial Avoider rats transitioned to a Non-Avoider phenotype and 32% of initial Non-Avoider rats shifted to an Avoider phenotype. These phenotypic shifts emerged as systematic, behaviorally meaningful subgroups when posthumously reassessed using an established criterion.

It is possible that aspects of the experimental design used in the present study contributed to the phenotypic shifts observed here, including the vendor source of the Wistar rats and the acoustic startle test, but are not reported in earlier work (Edwards et al., 2013). One potential factor is the acoustic startle test administered at Day 2 post-stress. Although this assay is sensitive to early stress-related alterations, it may also function as an additional stressor capable of influencing long-term behavioral outcomes, particularly in animals initially classified as Non-Avoiders. Consistent with this possibility, prior studies have identified an early post-stress window (*e.g.*, 2 days post-stress) during which PO-exposed rats can be distinguished statistically from unstressed controls, but not reliably differentiated into Avoider and Non-Avoider phenotypes (Albrechet-Souza et al., 2020; Roltsch et al., 2014). Thus, additional stress exposure during this early period may have influenced subsequent phenotypic classification in the present study. Whether secondary stressors, such as acoustic startle testing, increase vulnerability to PTSD-relevant outcomes remains an open question requiring further investigation. A second potential source of variability is the strength and potency of the bobcat urine at the time of exposure, which may influence the magnitude of stress induction, subsequent performance across anxiety-related behavioral assays, and the persistence of behavioral phenotypes within the PO CPA model. Consistent with this possibility, prior work has demonstrated positive relationships between PO concentration–including 2,3,5-trimethyl-3-thiazoline (TMT), a component of fox odor that is often isolated for PO studies (Rosen et al., 2015) and is known to elicit robust defensive responses, as well as odors derived from urine, fur, and feces–and the magnitude of defensive behaviors in rats (Blanchard et al., 2003; Brachetta et al., 2016; Wallace and Rosen, 2000). In the present study, potential dilution effects were minimized by providing fresh bobcat urine at the time of exposure after every two rats; however, variability in effective odor potency may still arise from factors such as urine composition (and other supply factors), volatility, or individual differences in olfactory sensitivity. Lastly, while multiple behavioral assays are commonly used across rodent PTSD models, to our knowledge no studies have examined hallmark anxiety-related behaviors and contextual conditioning longitudinally across multiple post-stress time points within the same animals. In the present study, we did not observe phenotype-dependent differences in standard anxiety-like behavior measures in either the open field or elevated plus maze, despite clear group differences from the CPA procedure (*e.g.*, time spent in the PO-paired chamber). As such, the present multi-assay design may have enhanced sensitivity to delayed or evolving behavioral phenotypes, while also introducing additional variables that may affect the interpretation of long-term outcomes. Collectively, the present findings do not undermine the validity of the PO model, as a subset of rats clearly exhibit long-lasting contextual conditioning in this experimental design. Rather, they suggest that behavioral phenotypic classification may change over time in some individuals and highlight the importance of systematically evaluating phenotype stability across extended post-stress intervals in future studies.

Additionally, we assessed longitudinal changes in body weight, along with circulating corticosterone levels and adrenal gland mass, as commonly used biomarkers of stress. Prior studies consistently demonstrated early stress-related physiological changes following the PO CPA procedure–most notably, reduced body weight gain shortly after stress (1 and 7 days post-stress) and altered HPA-axis signaling (*e.g.*, inverse relationships between corticosterone and avoidance behavior), without lasting changes in adrenal gland weight (Albrechet-Souza et al., 2020; Weera et al., 2020). These results suggested that early metabolic and endocrine responses occur rapidly after stress exposure but did not necessarily translate into sustained adrenal hypertrophy. In contrast, the current study found that sustained susceptibility was associated with a divergent physiological trajectory, characterized by increased rather than decreased body weight gain, beginning shortly after stress exposure and persisting across the 6-weeks period. The absence of significant differences in circulating corticosterone between sustained and shifted phenotypes, combined with unchanged adrenal gland weight, suggested that persistent behavioral susceptibility may not be driven by gross alterations in basal HPA-axis output, but instead by more subtle or context-dependent HPA-axis dysregulation. It is also possible that due to the longitudinal nature of this study, terminal timepoints are too far removed from the sensitive window for these biomarkers to demonstrate differences within this model. It is possible that long-term stress vulnerability reflected physiological adaptations in the brain (*e.g*., metabolic, receptor-level, or signaling pathway changes, as previously reported) (Weera et al., 2020; Whitaker et al., 2016), which should be evaluated in future studies in this context.

Lastly, despite the lack of clear visual clustering in the PCA plots, PERMANOVA identified significant differences between specific phenotype pairs, including Non-Avoiders, Avoiders, and transitioning groups, suggesting the emergence and persistence of anxiety-like behavior across Day 1 and 6 weeks post-exposure. Differences among stress-related phenotypes were better captured by variation along specific multivariate axes rather than by overall behavioral magnitude. In particular, the separation observed along PC2 from PCA with CPA-related behavioral features indicates that this PC2 component captures behaviorally meaningful variation linked to stress adaptation across repeated CPA testing, whereas PC1 appears to reflect variance largely shared across groups. However, when PCA was performed with the combined behavioral and physiological dataset, univariate comparisons of individual PC scores between phenotype pairs were mostly non-significant based on Mann–Whitney U tests. This suggests that pooling heterogeneous features can dilute phenotype-specific behavioral signals. In contrast, significant multivariate differences were observed between Non-Avoiders and Avoiders, highlighting the greater sensitivity of PERMANOVA for detecting coordinated behavioral changes that may not be evident along single dimensions alone (Anderson, 2001). Overall, these findings emphasize the value of focused behavioral feature sets and multivariate analytical frameworks for resolving subtle differences among stress-related phenotypes.

Several limitations of this study should be considered when interpreting the findings. First, the longitudinal trajectory analyses were performed retrospectively based on avoidance phenotypes determined at 6 weeks post-stress and should therefore be regarded as exploratory rather than hypothesis driven. Although a subset of animals transitioned between avoidance phenotypes over time, the present study cannot determine whether these changes reflect the natural evolution of stress-related behaviors, contextual learning, extinction-like processes, repeated behavioral testing, measurement variability, or interaction among these factors. Consequently, the observed transitions should be interpreted as changes in predator odor avoidance behavior rather than definitive evidence of changes in an underlying biological PTSD-relevant phenotype.

Second, while multiple behavioral assessments were conducted over the 6-weeks period following PO exposure, physiological measures of hypothalamic-pituitary-adrenal (HPA) axis function, including plasma corticosterone concentrations and adrenal gland mass, were assessed only at the terminal 6-weeks time point. Although no significant differences were detected between control and predator odor avoidance groups or among the avoidance phenotypes, this single terminal assessment does not provide a comprehensive evaluation of long-term HPA axis function. Factors including circadian variation, pre-stress basal endocrine status, stress-evoked hormonal responses, and repeated longitudinal measurements were not evaluated and may influence HPA axis activity. Therefore, future studies incorporating repeated endocrine assessments across multiple time points will be necessary to more comprehensively characterize long-term HPA axis function and stress reactivity in relation to the PO avoidance phenotypes. Accordingly, our findings should not be interpreted as evidence that corticosterone or adrenal gland mass lack utility as PTSD-relevant biomarkers, but rather that no differences were detected under the experimental conditions and at the time point examined in the present study.

Third, although animals were selected using a body weight criterion (200–275 g), there was considerable variation in the age of the rats at the time of predator odor exposures. Developmental stage has been shown to influence both behavioral and biological response to stress in rodents (Klein and Romeo, 2013; Romeo et al., 2016; Wah et al., 2019). Consequently, the inclusion of animals spanning approximately mid to late adolescence may have contributed to variability in the behavioral and physiological outcomes observed in this study. Future studies should employ a narrower age range to better control for developmental differences in stress reactivity.

Finally, only male rats were included in this study. Sex is a well-established biological variable in PTSD, with females exhibiting approximately twice the lifetime prevalence of PTSD and evidence of sex-specific differences in stress responsivity, neuroendocrine regulation, fear learning, and extinction (Mancini et al., 2025; Pooley et al., 2018). Consequently, the generalizability of our findings to females remains unknown. Future studies incorporating both sexes will be important for determining whether longitudinal changes in predator odor avoidance and associated behavioral and physiological responses differ as a function of sex.

Together, these findings advance our understanding of how PO exposure shapes long-term behavioral trajectories following traumatic stress. By combining automated behavioral quantification with longitudinal assessments of contextual conditioning, anxiety-related behavior, and peripheral stress biomarkers, the present study demonstrated that predator odor avoidance phenotypes are not entirely stable over extended post-stress intervals, with a subset of animals transitioning between avoidance phenotypes over time. More broadly, these results underscored the need for multi-assay, multi-time-point designs that incorporated both behavioral and physiological endpoints, as well as advanced analytic approaches, to systematically evaluate phenotypic stability and the factors that precipitate phenotypic shifts. Delineating the underlying neural mechanisms of persistent and delayed vulnerability versus resilience will be a critical next step for improving the translational utility of PO based CPA models and for informing strategies to prevent or reverse chronic stress related pathology. Future studies integrating longitudinal behavioral assessments with repeated endocrine, physiological, and molecular measurements will be important for determining whether changes in avoidance classification reflect alterations in stress susceptibility, extinction-like learning, contextual memory processes, or other biological adaptations following predator odor exposure.

## Data Availability

The original contributions presented in this study are included in the article/Supplementary material, further inquiries can be directed to the corresponding authors.
