# Supplementary material for: Longitudinal shifts in avoidance-based phenotypes in a predator odor conditioned place aversion model of posttraumatic stress disorder
Source: Front Behav Neurosci. 2026 Aug 25;20:1907963. doi: 10.3389/fnbeh.2026.1907963 (PMC13550178; doi:10.3389/fnbeh.2026.1907963)
Supplement: Supplementary file 1 [file Supplementary_file_1.docx]

Supplementary Material

# 1 Supplementary Figures and Tables

 Supplementary Figure 1. **Assessment of Anxiety-like behavior after CPA.** Control (grey bar, n = 21), Avoiders (yellow bar, n = 20), and Non-Avoiders (blue bar, n = 30) (black dots) were assessed for their acoustic startle response **(A)** at 2 days post-stress. The bar graph shows startle responses to 90, 105, and 115 dB, expressed as Vmax normalized by body weight (b.w.) in kilograms (Vmax/kg b.w.). The same groups of animals were evaluated for anxiety-like behavior in the open field **(B)**, measured as percent time spent in the center zone at 5 days post-stress, and in the elevated plus maze **(C)**, measured as percent time spent in the open arms at 16 days post-stress. Data are presented as mean ± SEM. For (A), statistical analysis was conducted using repeated-measures two-way ANOVA with sound level as the within-subject factor and experimental group (Control, Avoiders, and Non-Avoiders) as the between-subject factor, followed by Tukey's post-hoc test. Statistical significance is indicated for comparisons across sound intensity, where ***p* < 0.01 and *****p* < 0.0001. For (B) and (C), one-way ANOVA with Tukey's post-hoc test was used to compare between experimental groups. No statistically significant differences were observed among groups.

 **Supplementary Figure 2.** **Re-test to stressor at 6-weeks phenotype based on Day 1 post-stress phenotype classification. (A)** The bar graphs show the mean ± SEM time spent in the predator odor (PO)-paired chamber during the re-test session at 6-weeks post-stress. Red dotted line indicates the -10 s cut off score for classification of the PO phenotypes at Day 1 post-stress. **(B)** Gross locomotor activity is also reported for total distance traveled and average speed during the 5-min test at Day 1 post-stress. **(C)** Chamber metrics for the PO-paired context include number of entries, distance traveled, and travel speed. Data are shown as mean ± SEM for control (n = 22), Avoiders (n = 20), and Non-Avoiders (n = 30).

**Supplementary Figure 3.** **Automated Tracking Analysis of the sustained and shifted PO phenotypes at 1 day and 6-weeks post-stress.** Representative track plot **(i)** and respective heat map **(ii)** of control rats, sustained Avoider rats, Non-Avoider rats that shifted to the Avoider phenotype (Non-Avoiders **→** Avoiders), sustained Non-Avoider rats, and Avoider rats that shifted to the Non-Avoider phenotype (Avoiders **→** Non-Avoiders). Movement tracks are shown in magenta. For heatmaps, the hottest area (rainbow scale, red = maximum), represents the location where the animal spends the most time, and cool colors where the animal spends less time. PO-paired chamber is shown by the red line. Track plots and heatmaps of the same animals 6 weeks earlier, at the Day 4 of the CPA procedure, are shown above for comparison.

**Supplementary Figure 4.** **Human validated automated tracking-derived freezing episodes and time spent freezing at 6 weeks post-stress.** The bar graphs show the mean ± SEM of individual freezing episodes, during the 6-week retest **(A)**. Apparatus-wide total time spent freezing during the 6-week retest is shown in **(B)**. For both graphs, data are shown separated by 6-week collective diagnosis for the 6W timepoint, with control (n=22), Avoiders (n=12), Non-Avoiders→Avoiders (n=16), Non-Avoiders (n=14), and Avoiders→Non-Avoiders (n=8) as groups. Freezing episodes were identified first by ANY-maze and then validated by human raters using a Pearson correlation for IRR (*r* = 0.686) for the 6W timepoint. Time spent freezing was determined from the validated video timestamps. Analysis of both using a Kruskal-Wallis test with Dunn’s multiple comparisons post-hoc test revealed no statistically significant differences between groups in either dataset.

Supplementary Table 1. **Descriptive statistics for open field behavioral measures. Data are presented as mean ± SEM for each experimental group.**

Supplementary Table 2. **Descriptive statistics for elevated plus maze behavioral measures. Data are presented as mean ± SEM for each experimental group.**
